# A novel prognostic biomarker CD3G that correlates with the tumor microenvironment in cervical cancer

**DOI:** 10.3389/fonc.2022.979226

**Published:** 2022-09-13

**Authors:** Jingshuai Wang, Xuemin Gu, Leilei Cao, Yiqin Ouyang, Xiao Qi, Zhijie Wang, Jianjun Wang

**Affiliations:** ^1^ Department of Obstetrics and Gynecology, Shanghai East Hospital, Tongji University School of Medicine, Shanghai, China; ^2^ Department of Obstetrics and Gynecology, Tongji Hospital of Tongji University, Shanghai, China; ^3^ Department of Obstetrics and Gynecology, Shanghai Eighth People’s Hospital, Shanghai, China

**Keywords:** CD3G, cervical cancer, tumor microenvironment, ImmuneScore, scRNA, HPV

## Abstract

Cervical cancer (CESC) is the fourth most common and death-causing gynecological cancer, mostly induced by infection of human papillomavirus (HPV). Multiple components of the tumor microenvironment (TME), such as tumor infiltrating immune cells, could be targets of immunotherapy for HPV-related CESC. However, little is known about the TME of CESC until now. Here, we aimed to uncover the pathogenesis as well as to identify novel biomarkers to predict prognosis and immunotherapy efficacy for CESC. Combining the transcriptomic data and clinical characteristics, we identified differentially expressed genes in CESC samples from TCGA database by comparing the two groups with different ImmuneScore and StromalScore. Next, we detected ten key genes based on the PPI network and survival analyses with the univariate Cox regression model. Thereafter, we focused on CD3G, the only gene exhibiting increased RNA and protein expression in tumors by multiple analyses. Higher CD3G expression was associated with better survival; and it was also significantly associated with immune-related pathways through GSEA analysis. Furthermore, we found that CD3G expression was correlated with 16 types of TICs. Single cell RNA-sequencing data of CD3G in lymphocytes subgroup indicated its possible role in HPV defense. Hence, CD3G might be a novel biomarker in prognosis and immunotherapy for CESC patients.

## Introduction

In 2020, there were 604,000 cases of cervical cancer (CESC) leading to 342,000 deaths worldwide (1). CESC is now one of the most frequent cancers in females. The pathogenesis of CESC remains unclear until now, even though the treatment options including surgery, chemotherapy, and radiotherapy have been evolved over the past years. Up to 90% of CESC cases are made up of squamous cell carcinomas, which is the most common histological type (2); and about 95% of CESC are induced by persistent infection with carcinogenic human papillomavirus (HPV) (3). Nevertheless, HPV infection is considered a necessary but non-sufficient cause of CESC. Many other factors, including different signaling pathways, methylation, and genetic alterations, are involved in the tumorigenesis and development of CESC. Given the high incidence and mortality of CESC, uncovering the pathogenesis and identifying novel biomarkers are urgently demanded.

The tumor microenvironment (TME) is the surrounding ecosystem of the tumor, consisting of a variety of cell types and other non-cellular components. Studies over the past decades have shown that TME is an important determinant in the initiation and progression of various tumor types (4, 5). The immune system could recognize the viral oncogenes expressed by HPV-induced CESC, and thus control HPV infections during the progression of CESC. Given that the HPV-specific T regulatory cells may infiltrate cervical tumors and contribute to the TME establishment, immunotherapy could provide another option for the treatment of CESC (6). These treatments aim to enhance and/or activate the immunity of several tumor-infiltrating immune cells (TICs) (7, 8). Additionally, understanding the infiltration of immune cells will greatly help to guide clinical treatment of tumors (9). Recently, research on the TME has shown that the immune infiltration analysis and ImmuneScore could be jointly used to improve the prediction of cancer prognosis (10, 11). For example, the TME-related ImmuneScore in colorectal cancer is an effective indicator of metastasis and prognosis (11). Nevertheless, it remains unclear whether the interaction between CESC and the immune system could improve the prognosis of patients. Therefore, composition analysis of TICs in CESC will significantly help us understand the immune response to tumors and enhance immunotherapy in CESC.

In this study, we sought to identify the potential novel immune-related biomarkers for CESC through multiple bioinformatic analyses. To identify TME-related genes, we comprehensively used multi-omics data from several databases, including ImmuneScore, expression, clinical features, prognostic values, and methylation. We identified differentially expressed genes (DEGs) by analyzing transcriptomic data from the Cancer Genome Atlas (TCGA). GO enrichment, KEGG pathways and PPI network analyses were then used to discover the key genes. We finally confirmed CD3G as a potential biomarker, which might be used to predict prognosis in CESC.

## Materials and methods

### Acquisition of transcriptional data

The transcriptomic data and clinical information were downloaded from UCSC Xena database (cohort: TCGA TARGET GTEx, https://xenabrowser.net/). We then extracted the CESC data from TCGA-CESC for further analyses, including RNA-seq read counts, transcript per million reads (TPM) and methylation data. The immune infiltration data (including the ImmuneScore, StromalScore, and ESTIMATEScore) were downloaded from https://bioinformatics.mdanderson.org/estimate/ (12). There are 293 CESC samples with overall survival (OS) time > 1 day, which also contain clinical data and immune infiltration scores.

### Identification of TME-related DEGs

Using the median value of their ImmuneScore, StromalScore, or ESTIMATEScore as cutoff, the CESC samples were divided into two groups, high and low. We then performed the survival analysis and depicted the Kaplan–Meier curves using the “survival” and “survminer” R packages. The primary prognostic endpoint was defined as the OS, disease-specific survival (DSS) or progression-free interval (PFI) in the survival analysis. The two groups were compared with the likelihood ratio test.

We then screened the DEGs between the ImmuneScore or StromalScore groups using the “limma” R package (13). The cutoff threshold was defined as *p* < 0.05 and |log_2_(fold change)| > 1. The DEGs associated with OS were identified using the survival analysis with the univariate Cox regression model.

### GO and KEGG enrichment analyses

The functional significance of the DEGs was explored by two enrichment analyses in the “clusterProfiler” R packages (14): gene ontology (GO) terms and the Kyoto Encyclopedia of Genes and Genomes (KEGG) pathways. In both enrichment analyses, the statistically significant cutoff was defined as *p* < 0.05 and *q* < 0.05. The results were visualized in bubble and circos plots (15).

### Integration of PPI network

With the confidence score set to 0.9, the protein-protein interaction (PPI) network was constructed by applying the DEG-encoded proteins to the online STRING database (http://string-db.org) (16). We then transferred the network into Cytoscape for visualization (17). The modular analysis was performed to identify the hub genes using cytoHubba embedded in Cytoscape (18). Then, the multi-network clustering (MNC) method was used to screen the top 30 significant genes.

### Expression pattern of key genes

Since TCGA database does not contain enough normal tissue samples, we included the RNA sequencing data in TPM format from GTEx. The expression of key genes was then compared between normal tissues and primary tumors. To validate the expression pattern of key genes, we first obtained two array datasets: the array data of GSE63514 which includes 24 normal tissues and 28 cervical cancer tissues (Affymetrix, normalized GC-RMA values, log_2_ transformed). Moreover, this dataset additionally includes samples spanning three stages of cervical intraepithelial neoplasia (14 CIN1, 22 CIN2 and 40 CIN3) (19); and the array data of GSE7410 which includes five normal tissues and nine early-stage cervical tumors (Agilent) (20). Second, we obtained three sets of high-throughput RNA sequencing data: GSE87410 which includes paired samples (tumor and the adjacent normal tissue) from six CESC patients (21); GSE192804 which includes paired samples from 14 CESC patients; and GSE149763 which includes normal tissues and tumors with three biological replicates (22). We used kallisto (23) to calculate the expression level of each gene for GSE192804 because it provides the raw sequencing data. The immunohistochemistry (IHC) was used to determine the protein expression in normal tissues and tumors, which were obtained from the Human Protein Atlas (HPA) database (https://www.proteinatlas.org/).

CpG islands can potentially regulate gene expression by DNA methylation. There are several CpG sites close to the transcriptional start site of CD3G. We used probe cg15880738 in the 5’ UTR region of CD3G to evaluate the correlation between its expression level and methylation pattern.

There are 178 core-set TCGA-CESC samples with HPV genotype information: 169 HPV-positive, 120 alpha-9 types (103 HPV16, five HPV52 and five HPV58), 45 alpha-7 types (27 HPV18 and nine HPV45), and nine HPV-negative (24). The other HPV types with less than five samples were excluded from our analysis. The expression level of CD3G was examined in these groups.

### Gene set enrichment analysis

We used GSEA including the KEGG and Reactome pathways in the “clusterProfiler” R package to screen genes correlated with CD3G. The parameters were set as follows: nPerm = 1000, minGSSize = 5, maxGSSize = 5000, and *p* < 0.05 (14).

### Evaluation of immune cell infiltration

We used the immune infiltration scores calculated by CIBERSORT to estimate the proportions of infiltrating cells in the TME (25). The Wilcoxon rank sum test was performed to compare the difference between the two groups. Spearman’s correlation was applied to assess the association between the proportions and CD3G expression, and between tumor mutation burden (TMB) and CD3G expression.

### Single-cell RNA sequencing analysis

GSE168652 contains the scRNA-seq data from 14,220 cells of CESC and 11,422 cells of the adjacent normal tissues. The “Seurat (4.1.1)” R package was used to perform quality control and analysis of the scRNA-seq data. We filtered out the low-quality cells using the following criteria: < 200 genes expressed in a single cell; genes that were expressed in < 3 cells; and > 10% of the percentage of mitochondrion-derived genes.

The top 2,000 variable genes were used for principal components analysis (PCA). After the reduction of the dimensionality, the first 30 principal components were visualized by the t-Distributed Stochastic Neighbor Embedding (t-SNE) plots. Before clustering, we used the ScaleData function to regress the influence of the percentage of mitochondrion-derived genes.

All cells were divided into 15 clusters with resolution = 0.5, and further clustered into seven subgroups according to the published markers (26): cancer cells, endothelial cells, endometrial stromal cells, fibroblasts, lymphocytes, macrophages, and smooth muscle cells.

## Results

### Analysis process of datasets

**Figure 1:**
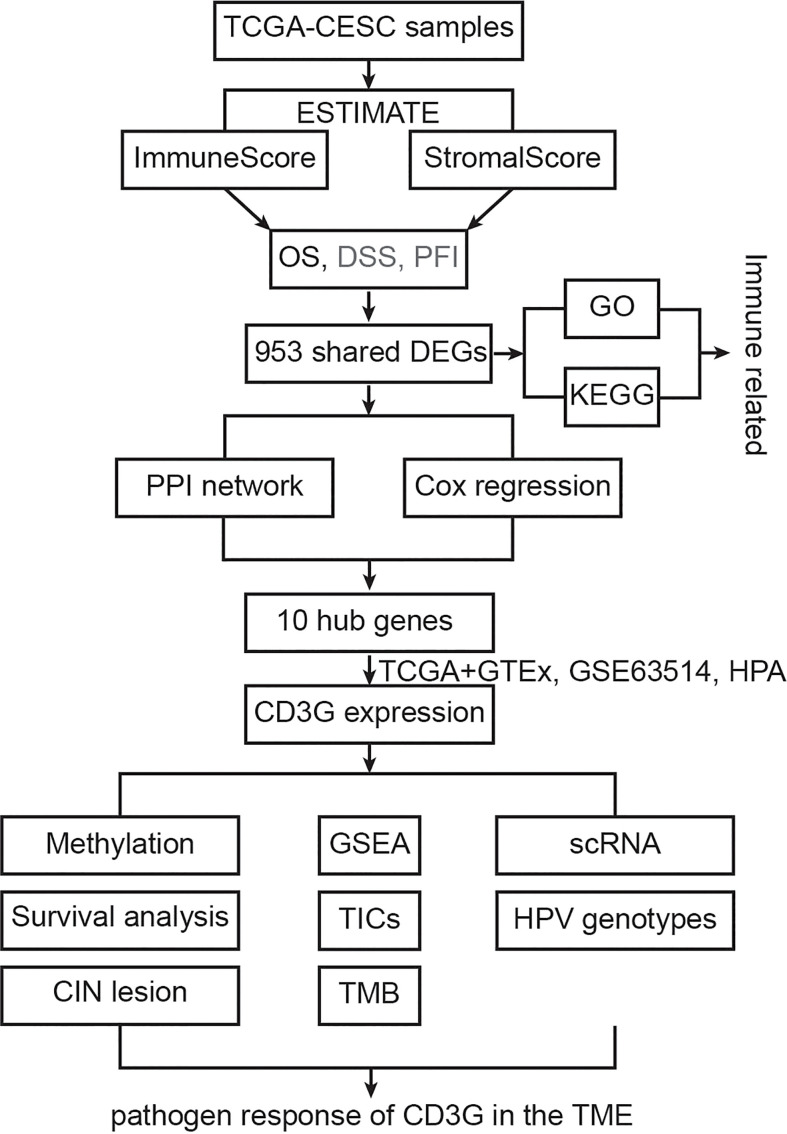
The flowchart showing the process of this study.

The study design was shown in a flowchart (**Figure 1**). The ImmuneScore and StromalScore were downloaded to assess the immune and stromal components. DEGs were identified between the two ImmuneScore groups, as well as two StromalScore groups. Next, the top 30 hub genes were screened out from DEGs using the PPI network analysis. Meanwhile, we performed the univariate Cox regression analysis to identify 215 significant genes. The intersection of the two datasets revealed ten key genes, i.e., ZAP70, CD3D, GRAP2, CD2, CD3G, CD8A, BTK, CD247, CD3E, and HCK. We then focused on CD3G, which had significantly higher RNA and protein expression in CESC tumors compared to the normal tissues. An integrated analysis was further performed on CD3G, which included methylation, survival, CIN lesion, GSEA, correlation with the proportions of TICs, TMB, single-cell subgroups, and HPV genotypes.

### Association of ImmuneScore with the prognosis of CESC

**Figure 2:**
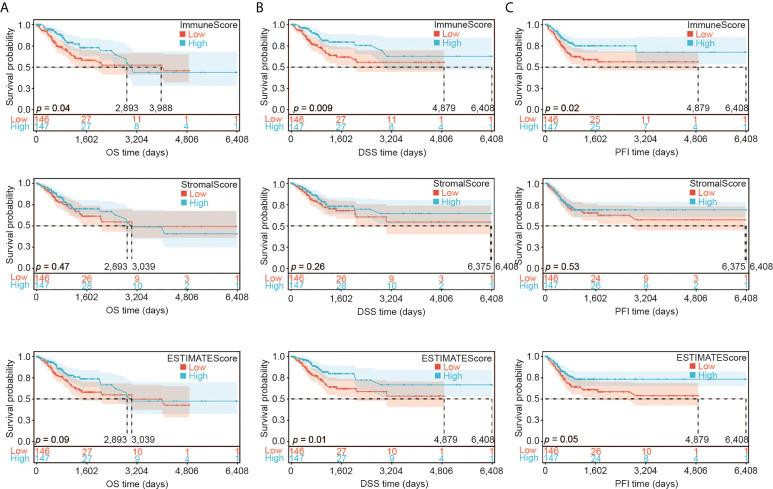
Association of ImmuneScore, StromalScore and ESTIMATEScore with CESC patients’ prognosis. **(A)** Overall survival (OS) analysis of ImmuneScore, StromalScore and ESTIMATEScore for CESC patients. **(B)** Disease special survival (DSS) analysis of ImmuneScore, StromalScore and ESTIMATEScore for CESC patients. **(C)** Progression free survival (PFI) analysis of ImmuneScore, StromalScore and ESTIMATEScore for CESC patients.

We first assessed the association of the immune infiltration scores with the prognosis of CESC patients. We divided the samples into two groups according to their median value of ImmuneScore, StromalScore or ESTIMATEScore (246.57, -1051.79, or -733.25, respectively). We then constructed the Kaplan-Meier survival curves to examine the difference of OS (**Figure 2A**), DSS (**Figure 2B**), and PFI (**Figure 2C**) between these two groups with high or low scores. Overall, better OS, DSS and PFI were found in samples with a higher ImmuneScore than in samples with a lower ImmuneScore (**Figure 2A**, likelihood ratio test *p* = 0.04, 0.009, and 0.02, respectively). StromalScore did not have a significant association with OS, DSS, or PFI (**Figure 2B**), while ESTIMATEScore had a significant association with DSS or PFI but not OS (**Figure 2C**). Overall, our results indicate that the immune components of TME may positively influence the prognosis of CESC patients.

### Most TME-related DEGs are involved in immune responses

**Figure 3:**
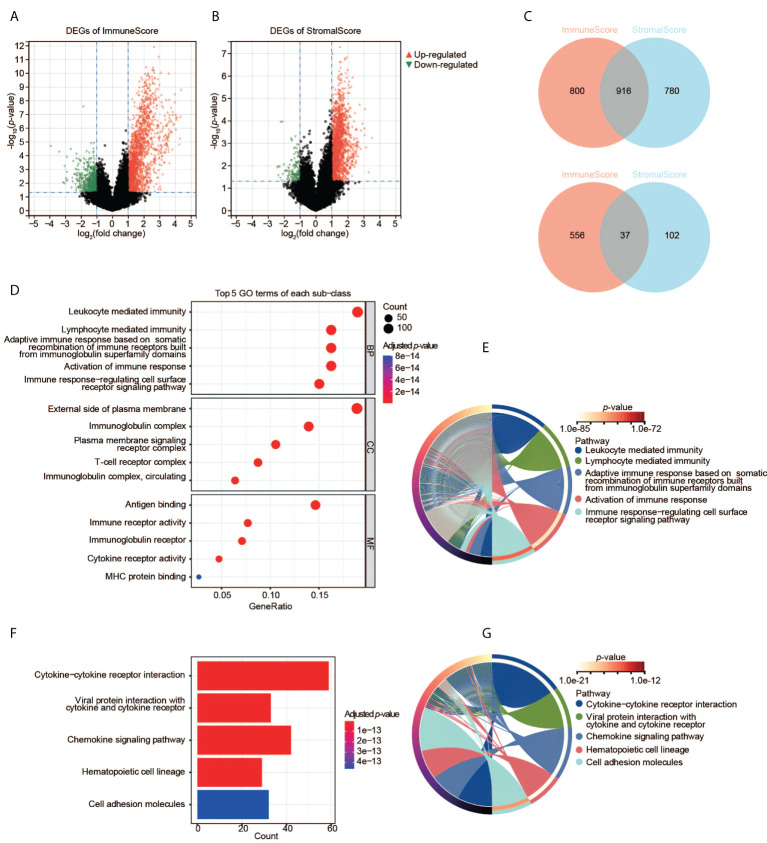
Identification and pathway enrichment analyses of DEGs. **(A)** DEGs based on the two groups with high and low ImmuneScore. **(B)** DEGs based on the two groups with high and low StromalScore. **(C)** Shared DEGs between the ImmuneScore and StromalScore analyses. Up-panel: upregulated DEGs; down-panel: downregulated DEGs. **(D)**, **(E)** GO enrichment analyses of DEGs. **(F)**, **(G)** KEGG enrichment analysis of DEGs.

We then analyzed the transcriptomic data of the TCGA-CESC samples. We found 2,309 DEGs in total between two ImmuneScore groups (high and low), including 1,716 upregulated and 593 downregulated genes (**Figure 3A**), and 1,835 DEGs between the two StromalScore groups (1,696 upregulated and 139 downregulated genes, **Figure 3B**). Moreover, 916 upregulated and 37 downregulated genes were shared among both analyses (**Figure 3C**), indicating that they were key factors affecting the TME status of CESC.

To understand the function of DEGs in the initiation and progression of CESC, we then performed GO and KEGG analyses. The 953 shared DEGs were enriched in the pathways related to the immune system. First, the enrichment analysis of GO terms revealed that the 953 shared DEGs were significantly clustered in immune-related categories (**Figure 3D**), such as leukocyte-mediated immunity, lymphocyte-mediated immunity, immunoglobulin complex, external side of plasma membrane, antigen binding, as well as immune receptor activity. The top five significant biological process (BP) terms were all related to immunity and immune responses (**Figure 3E**). Second, the enrichment analysis of KEGG showed that the top five pathways of these DEGs were also immune-related, such as cytokine-cytokine receptor interaction, etc (**Figures 3F, G**). Thus, these DEGs may be immune factors influencing the TME of CESC.

### PPI network and prognostic signature analyses

**Figure 4:**
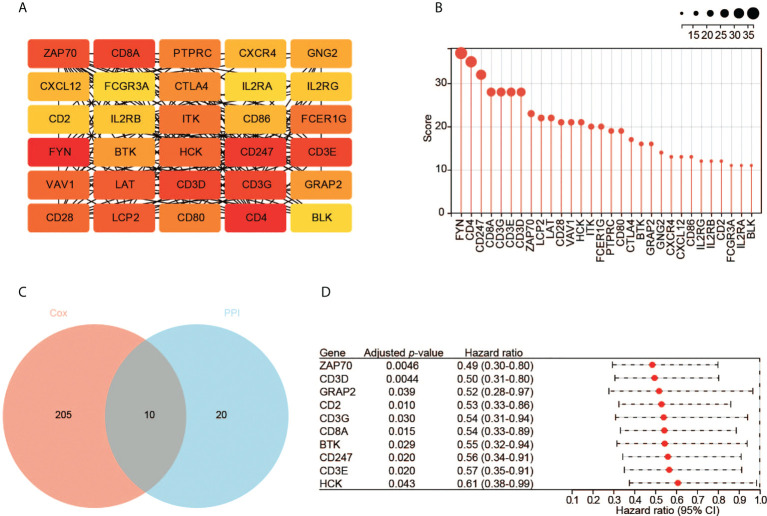
Identification of hub genes. **(A)** The top 30 hub genes based on the analysis of PPI network. Color from yellow to red represents the low to high MNC score. **(B)** The MNC scores of the top 30 hub genes. **(C)** The 10 key genes based on the interaction of the two datasets. **(D)** The survival analysis with the univariate Cox regression model. Here shows the 10 key genes.

We constructed the PPI network of the 953 DEGs using STRING. The PPI network contained 644 nodes and 769 edges. We then employed Cytoscape to analyze the interactive relationship of the candidate proteins (**Supplementary Figure S1**). By using the MNC algorithm in the cytoHubba, the top 30 hub genes were identified, which might have critical functions in CESC oncogenesis (**Figures 4A, B**).

Meanwhile, we performed the prognostic analysis of the 953 DEGs. In total, 215 significant genes were revealed by univariate Cox regression analysis (*p* < 0.05). Ten hub genes (ZAP70, CD3D, GRAP2, CD2, CD3G, CD8A, BTK, CD247, CD3E, and HCK) were found by the intersection of the two aforementioned datasets (**Figure 4C**). All these genes are protective factors with a hazard ratio < 1 (**Figure 4D**).

### Identification of CD3G as the key gene in CESC

**Figure 5:**
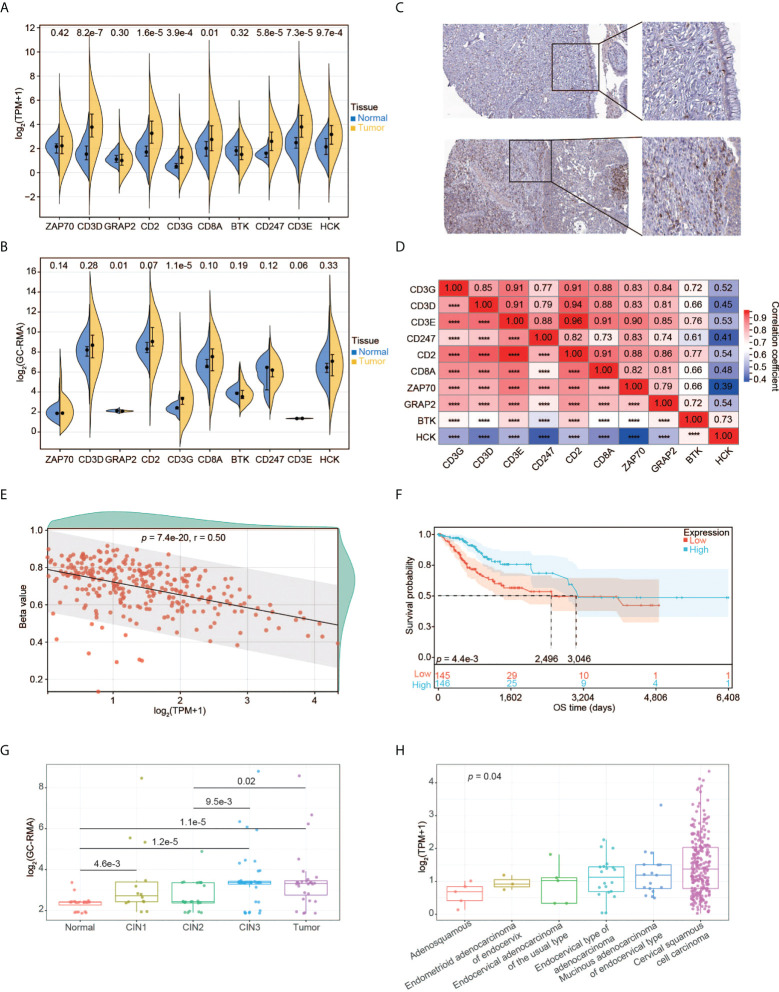
Expression pattern of CD3G as the key gene in CESC. **(A)** The expression profiles of the 10 key genes in normal tissues and tumors. **(B)** The expression profiles of the 10 key genes validated using GSE63514. **(C)** CD3G protein expression based on Immunohistochemistry data from the HPA database. Up panel: normal tissue. Down panel: CESC tissue. Antibody: HPA038494. The patient id of the normal cervix tissue is 1773. It has no detectable score of antibody staining. The patient id of the tumor tissue is 1751. It has medium staining, moderate intensity, and 75%-25% quantity score. **(D)** The co-expression of the 10 key genes in CESC. ****p < 0.0001. **(E)** The methylation pattern of CD3G in CESC. **(F)** OS analysis of high and low CD3G expression for TCGA-CESC patients. **(G)** The association of CD3G expression with CIN lesion. **(H)** The association of CD3G expression with histological types.

Using the RNA-seq data, we explored the expression pattern of the 10 hub genes. We found that CD3D, CD2, CD3G, CD8A, CD247, CD3E, and HCK had significantly higher expression in tumors relative to normal tissues (**Figure 5A**). We also validated the expression pattern of these genes using the gene expression profiles by array and high-throughput RNA-seq data from five different datasets. Compared to normal tissues, only CD3G was constantly significantly over-expressed in tumors (**Figure 5B** and **Supplementary Figures 2A-D**). Moreover, higher expression of CD3G protein was detected in tumors than normal tissues based on the IHC data from the HPA database (**Figure 5C**). Thus, CD3G was considered the key gene in CESC in our study.

Interestingly, CD3D, CD3E, CD3G, and CD247 (or CD3Z) are associated with T-cell immunity by forming the T-cell receptor-CD3 complex (TCR/CD3). The co-expression analysis indicated that these four genes as well as CD2 were highly correlated (**Figure 5D**). We then examined the association between methylation and the expression level of CD3G. The CpG methylation of CD3G was negatively correlated with its expression level (**Figure 5E**), suggesting that this CpG site is likely associated with the transactivation of CD3G.

We found significantly longer OS time of CESC patients with higher CD3G expression compared to those with lower CD3G expression (**Figure 5F**). During the multi-step process of cervical cancer development, CD3G expression was significantly increased in CIN1 lesions compared to normal tissues (**Figure 5G**). This change in CIN1 was maintained through progression to CIN2 and enhanced to CIN3 and cancer.

By dissecting the histological types of CESC samples, we found a significant difference in expression levels between different groups (**Figure 5H**, *p* < 0.05, Kruskal-Wallis rank sum test). Compared to other histological types, CD3G had the highest expression in Cervical Squamous Cell Carcinoma (CSCC). These patterns suggest a crucial role of CD3G in CESC progression.

### GSEA of CD3G

**Figure 6:**
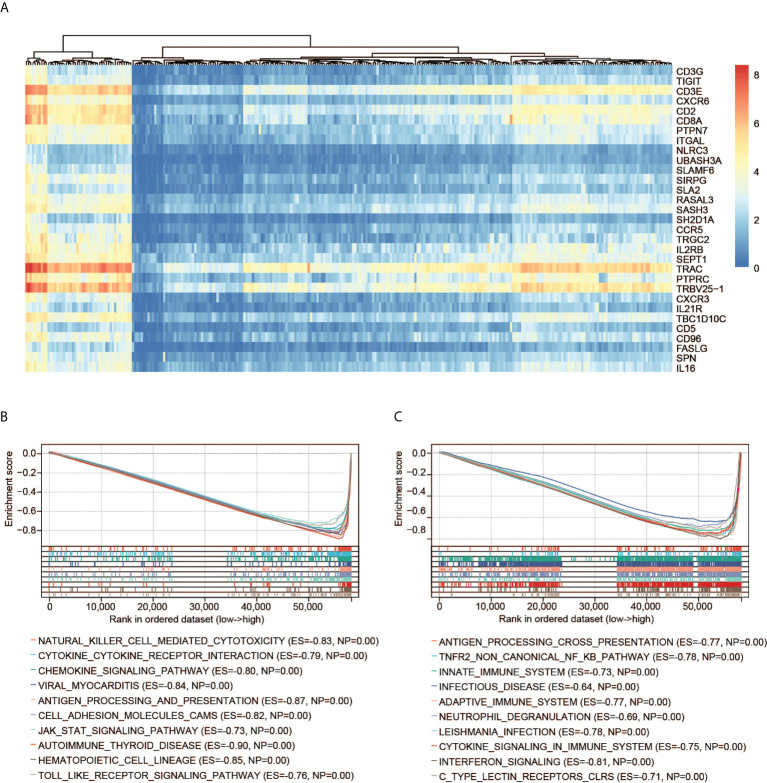
GSEA of CD3G. **(A)** The expression profiles of the top 30 genes contributing to core enrichment. **(B)** The top 10 significant KEGG pathways associated with high CD3G expression. **(C)** The top 10 significant Reactome pathways associated with high CD3G expression.

GSEA including the KEGG and Reactome pathways was performed to study the differences in groups with high and low expression of CD3G. The GSEA results showed that several highly correlated genes were immune-related (**Figure 6A**), indicating the important biological role of CD3G in the immune status of TME. The significantly enriched KEGG pathways were immune-related. The top five pathways are antigen processing and presentation, chemokine signaling pathway, cytokine-cytokine receptor interaction, natural killer cell-mediated cytotoxicity, as well as viral myocarditis (**Figure 6B**). Similarly, the significant Reactome pathways were also immune-related, including adaptive immune system, antigen processing cross-presentation, infectious disease, innate immune system, and TNFR2 non-canonical NF KB pathway (the top 10 pathways are shown in **Figure 6C**).

### Correlation of CD3G expression with TICs

**Figure 7:**
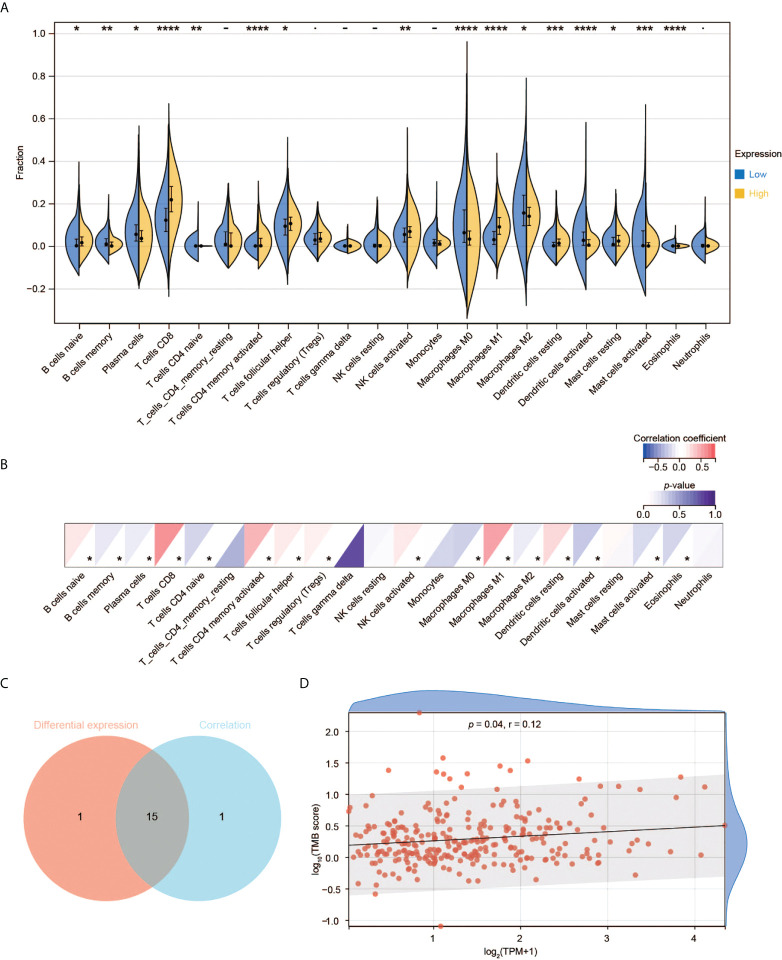
Immune cell infiltration analysis of CD3G. **(A)** The difference of TIC proportions with high or low CD3G expression in CESC. **(B)** The correlation of the TICs and CD3G expression. *p < 0.05, **p < 0.01, ***p < 0.001, ****p < 0.0001. **(C)** The shared TICs between the analyses in **(A, B)**. **(D)** The correlation of TMB and CD3G expression.

We used RNA-seq data from TCGA to perform CIBERSORT analysis and obtained the proportion of 22 immune cell types. We then investigated the interplay between CD3G expression and TME. Among the 22 types of TICs, 16 immune cell types had significant differences among the two groups with high and low CD3G expression (**Figure 7A**). In the correlation analysis, we found that eight types of TICs were positively correlated with CD3G expression, while another eight types had a negative correlation to CD3G expression in CESC samples (**Figure 7B**). The intersection of difference and correlation analyses revealed 15 shared types of TICs (**Figure 7C**). We also found a positive correlation of CD3G expression with TMB (**Figure 7D**, r = 0.12, *p* = 0.04).

### Specific expression of CD3G in lymphocytes against HPV

**Figure 8:**
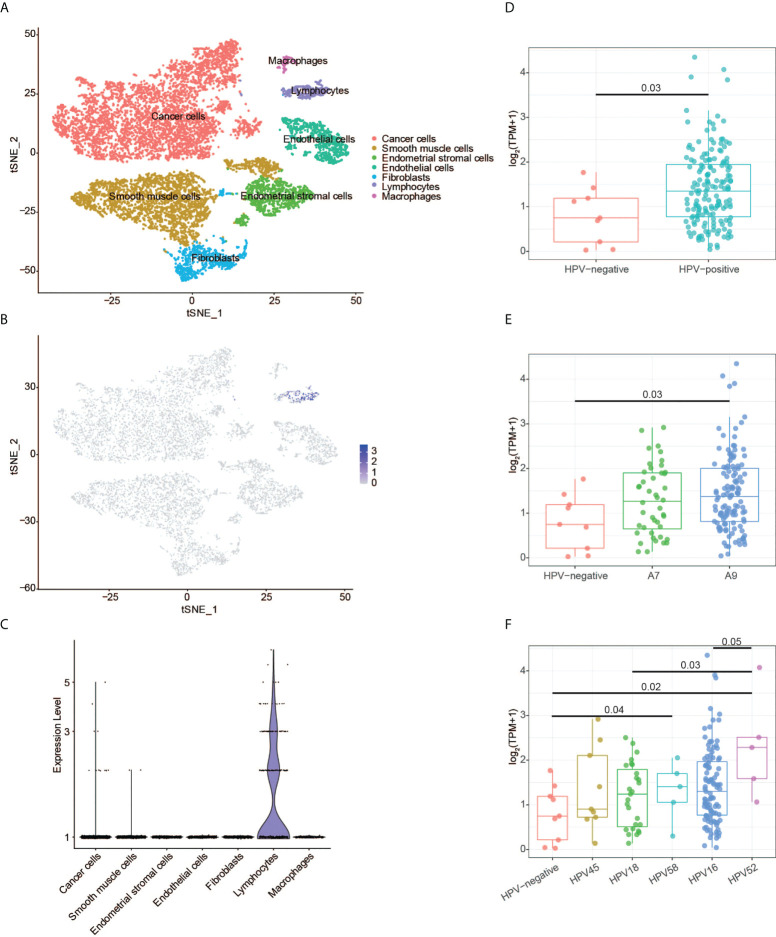
CD3G expression revealed by single-cell RNA sequencing and HPV genotype analyses. **(A)** The t-SNE plot of cells from the normal tissues and cervical cancer. **(B)** The t-SNE plot showing the CD3G expression. **(C)** The enrichment of CD3G in lymphocytes subgroup in cervical cancer. **(D-F)** The correlation of CD3G expression and HPV types.

The scRNA-seq data of the normal tissues and CESC were used to perform t-SNE analysis. We identified 15 cell clusters (**Supplementary Figure 3**) and annotated them into seven cell subgroups with reported markers: cancer cells, endothelial cells, endometrial stromal cells, fibroblasts, lymphocytes, macrophages, and smooth muscle cells (**Figure 8A**). CD3G was mainly expressed in lymphocytes subgroup in the t-SNE plot (**Figure 8B**). Meanwhile, CD3G was enriched in lymphocytes subgroup when the relative expressions of CD3G in each subgroup were determined (**Figure 8C**). These results were not surprising since CD3G is one member of the TCR/CD3 complex.

Next, we sought to find the possible function of CD3G in CESC. It has been reported that T-cells, especially CD4 T-cells have HPV-specific responses in CESC (27). We extracted the HPV-positive and -negative CESC samples and investigated the expression pattern of CD3G in these groups. Compared to the HPV-negative samples, HPV-positive samples had a significantly higher expression level of CD3G (**Figure 8D**). After dividing HPV into A7 and A9 genotypes, we found that the higher expression level of CD3G was more significant in A9 but not A7-infected samples compared to HPV-negative samples (**Figure 8E**). A7 types can be further divided into HPV18 and HPV45, and A9 types can be divided into HPV16, HPV52, and HPV58. We found CD3G was highly expressed in HPV52-infected samples (**Figure 8F**). Therefore, as a member of TCR/CD3 complex, CD3G may play a defensive role against different types of HPV in the TME of CESC.

## Discussion

As the fourth most common tumor in women, CESC leads to high morbidity, especially in developing countries. Discovering prognostic genes involved in the TME will benefit early diagnosis and management of CESC. Here, we performed comprehensive analyses and sought to identify novel biomarkers in CESC. CD3G was potentially a prognostic and therapeutic biomarker in CESC, which was associated with the immune responses.

The ESTIMATE algorithm can help to identify candidate TME-related biomarkers since it is an integrated approach to estimate tumor purity based on gene expression. We used this algorithm to identify ImmuneScore and StromalScore of CESC samples based on TCGA transcriptomic data. Consistent with previous reports that the factor related to the TME could potentially be used to estimate prognosis (28, 29), we observed a correlation between higher ImmuneScore and better OS, DSS and PFI, suggesting TME composition would influence the clinical outcomes of CESC patients. The identified TME-related DEGs were found to be related to immune responses using GO and KEGG enrichment analyses. Our results indicate the functions of immune cells and stromal cells are interrelated in establishing TME in CESC.

Combining the PPI network and survival analyses, we revealed 10 key genes, comprising ZAP70, CD3D, GRAP2, CD2, CD3G, CD8A, BTK, CD247, CD3E, and HCK. Further analysis demonstrated that ZAP70, GRAP2, and BTK did not show a higher expression level in tumors than in normal tissues. We confirmed that CD2 protein had a low score of antibody staining, while CD3G and HCK protein had a medium score of antibody staining in the HPA database. The proteins of CD3D, CD3E, CD3G, and CD247 are expressed on the surface of T-cells to form the TCR/CD3 complex. This complex has anti-tumor activity by coupling tumor-associated antigen recognition to intracellular signaling initiation (30, 31). We found that CD3D, CD3E, CD3G, CD247, as well as CD2 were highly co-expressed in CESC (**Figure 5D**). Such a result is consistent with the interaction between CD2 protein and TCR/CD3 complex in T-cell activation (32). However, they also have their own unique functions in addition to the role of signal transduction. We could only detect the RNA and protein expression changes of CD3G, but not CD3D, CD3E or CD247 between tumor and normal tissues. Hence, we selected CD3G for further investigation.

Previous studies have shown that CD3D may serve as a prognostic maker of colon cancer, gastric cancer and other tumors (33, 34); CD3E can be used as a prognostic factor of lung cancer (35); the expression of CD247 was associated with many cancers, including gastric cancer, head and neck cancer and ovarian cancer (36–38). Very few studies mentioned that CD3G was related to the initiation of CESC or the other cancer types. Compared to normal tissues, higher CD3G expression was found in CESC samples associated with a better prognosis. These results are consistent with the other studies using different methods in CESC (39, 40). However, as one of the key genes, CD3G has not been thoroughly analyzed or defined as a biomarker for its association with CESC in these studies. According to our knowledge, it is the first time to screen CD3G as an important prognostic marker in CESC using the ESTIMATE algorithm.

During the multi-step process of CESC development, the expression of CD3G was significantly increased starting from CIN1 lesion to cancer. We also found CD3G had the highest expression in the histological type of CSCC. These patterns suggest CD3G takes a part in the progression of CESC. Higher TMB indicates better immunotherapy efficacy in multiple clinical studies of tumors (41–43). The positive correlation between the CD3G expression and the TMB in our study provides new insights into immunotherapy for CESC.

Furthermore, high expression of CD3G expression was also associated with immune-related pathways as revealed by the GSEA analysis, suggesting that the CD3G expression in CESC patients may influence the TME. As one of the important immunoreceptors, CD3G was specifically expressed in lymphocytes of CESC patients. Thus, it may initiate the adaptive immune response in the TME of CESC. Accordingly, we found that CD3G was specifically expressed in lymphocytes by analyzing scRNA data of CESC. Moreover, CD3G of HPV-positive samples was significantly over-expressed compared to the HPV-negative samples, and it had a higher sensitivity to prevent HPV52, which belongs to the A9 type. One possible explanation is that CD3G could recognize the viral oncogenes expressed by HPV-induced CESC. Interestingly, we found that 16 types of TICs were significantly correlated to the expression of CESC. Therefore, our results demonstrated the important role of CD3G in the TME of CESC.

A few studies showed that a favorable prognosis was indicated by the dense infiltration of certain immunocytes (CD8 and CD4 T-cells) (44, 45). Recently, one study showed that T-cells in the CD3G-deficient patients had reduced diversity, reduced suppressive function, and increased clonality in autoimmunity (46). Another study showed that CD3G was upregulated after treatment with reovirus in TRP1 tumors (47). In addition, It has been reported that after neoadjuvant immunotherapy with the IRX-2 regimen, the association of CD3G upregulation with increased immune infiltration could be observed in the TME of head and neck epithelial tumors (48). These studies, including ours, revealed the functional pathogen response of CD3G in the TME. After examining the co-expression pattern of CD3G and 60 immune checkpoints, we found that CD3G was positively correlated to several important inhibitory and stimulatory genes in CESC (**Supplementary Figures 4A, B**), such as TIGIT, PDCD1, OCOS and PRF1. Therefore, CD3G may be a potential therapeutic target in immunotherapy. Further investigations are required to fully understand the mechanisms of CD3G-induced immune activities.

In conclusion, our observations collectively demonstrated that as a prognostic marker of CESC, CD3G may guide the development of immunotherapy. In particular, CD3G may have the immunomodulatory effects to delay disease progression. Thus, CD3G deserves more exploration because of its potentiality in diagnosis, prognosis, and therapeutic targets for CESC. Further experiments are required to verify our results based on bioinformatic analyses.

## Data availability statement

The original contributions presented in the study are included in the article/**Supplementary material**. Further inquiries can be directed to the corresponding authors.

## Author contributions

This study was designed by JJW and ZW. JJW and JSW wrote the manuscript. ZW reviewed the manuscript. JSW, XG, and LC performed the data analysis. YO analyzed the single-cell RNA-seq data. XQ helped to generate the images. All authors contributed to the article and approved the submitted version.

## Funding

This work is supported by the Shanghai Xuhui District Medical Peak Subject Project (SHXH201713).

## Acknowledgments

We thank the medical project of Xuhui District Peak Subject to support this work (SHXH201713).

## Conflict of interest

The authors declare that the research was conducted in the absence of any commercial or financial relationships that could be construed as a potential conflict of interest.

## Publisher’s note

All claims expressed in this article are solely those of the authors and do not necessarily represent those of their affiliated organizations, or those of the publisher, the editors and the reviewers. Any product that may be evaluated in this article, or claim that may be made by its manufacturer, is not guaranteed or endorsed by the publisher.
