# Supplementary material for: A novel prognostic biomarker CD3G that correlates with the tumor microenvironment in cervical cancer
Source: Front Oncol. 2022 Sep 13;12:979226. doi: 10.3389/fonc.2022.979226 (PMC9513466; doi:10.3389/fonc.2022.979226)
Supplement: Supplementary file 1 [file DataSheet_1.pdf]

Supplementary Material

Supplementary Figures

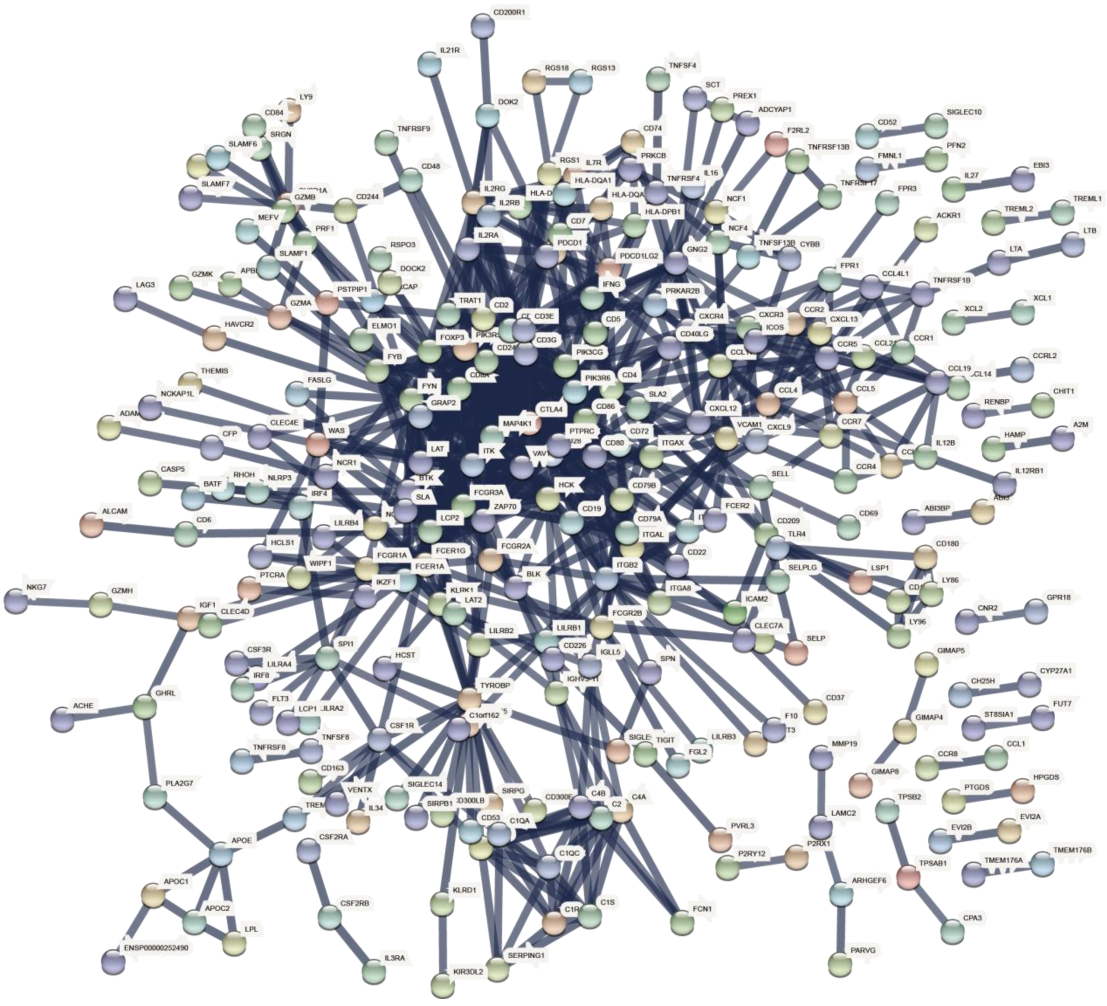

Supplementary Figure 1. PPI network based on STRING confidence score > 0.9.

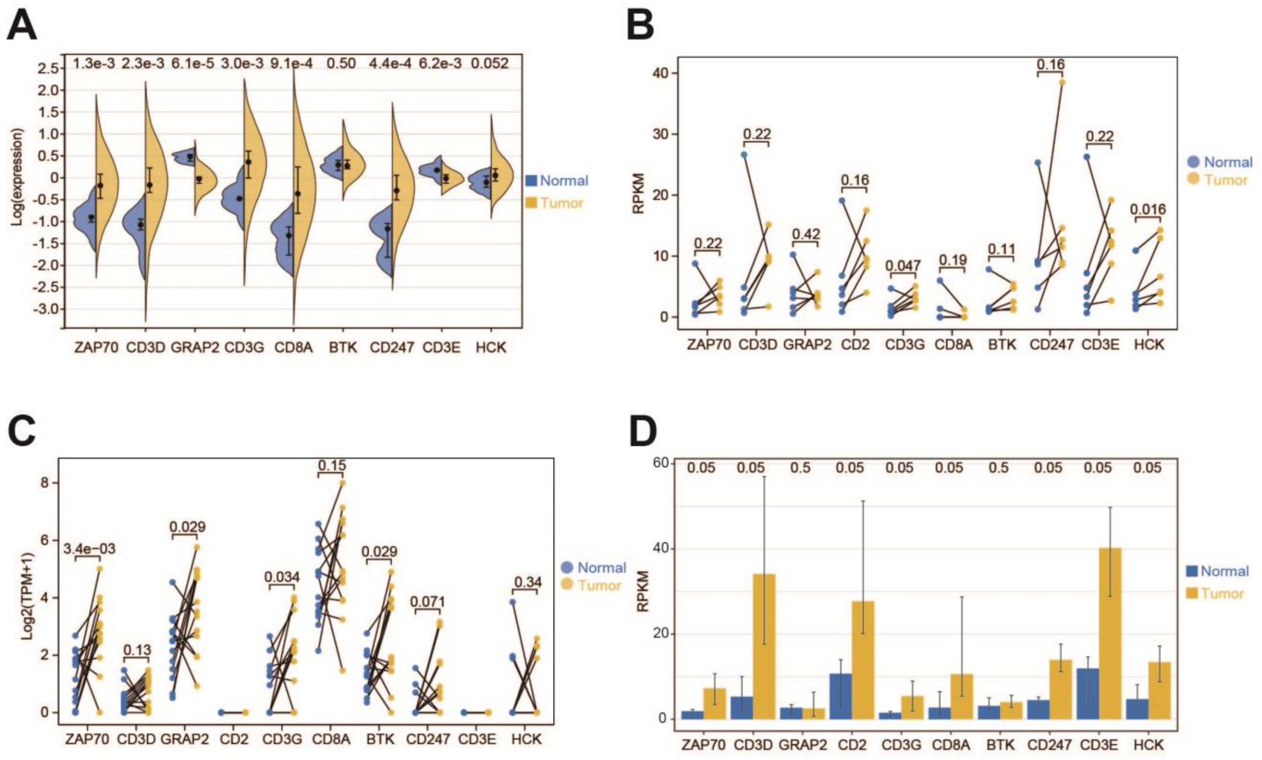

Supplementary Figure 2. Validation of the expression of CD3G as the key gene in CESC. (A) The expression profiles of the key genes using GSE7410. (B) The expression profiles of the key genes in paired tumor and adjacent normal tissues using GSE87410. (C) The expression profiles of the key genes in paired tumor and adjacent normal tissues using GSE192804. (D) The expression profiles of the key genes using GSE149763.

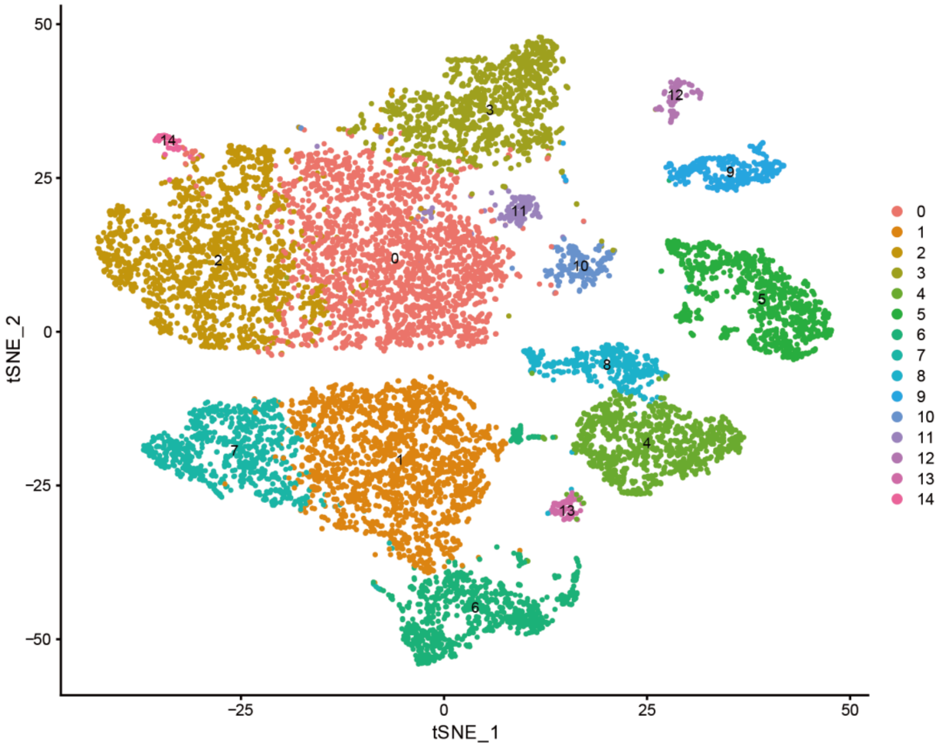

Supplementary Figure 3. The t-SNE plot of cells with each cell color-coded for associated cell type.

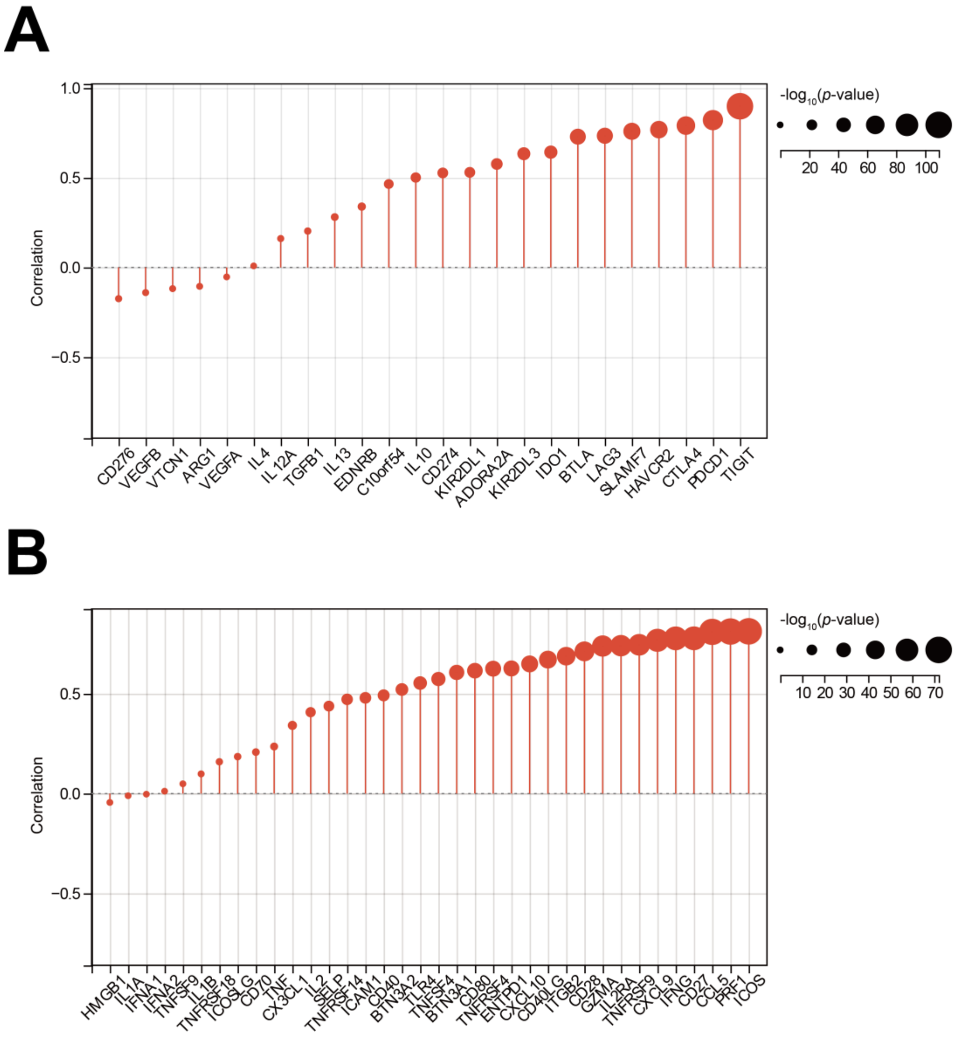

Supplementary Figure 4. The correlation of CD3G and immune checkpoints. (A) CD3G was correlated to immune inhibitory genes. (B) CD3G was correlated to immune stimulatory genes.

Supplementary Tables

Supplementary Table 1. The clinical information of the CESC patients.

ID	OS_time	OS	DSS_time	DSS	PFI_time	PFI	Immune_score	Stromal_score	ESTIMATE_score
TCGA-2W-A8YY-01	533	0	533	0	533	0	-317.99	-1244.22	-1562.21
TCGA-4J-AA1J-01	542	0	542	0	542	0	-373.13	-837.06	-1210.2
TCGA-BI-A0VR-01	1505	0	1505	0	1505	0	1072.14	-709.45	362.69
TCGA-BI-A0VS-01	1735	0	1735	0	1735	0	286.23	-579.33	-293.09
TCGA-BI-A20A-01	720	0	720	0	720	0	997.66	-844.39	153.27
TCGA-C5-A0TN-01	348	1	348	1	62	1	1.68	-1254.11	-1252.43
TCGA-C5-A1BE-01	2094	1	2094	1	1674	1	4.89	-1137.9	-1133.01
TCGA-C5-A1BF-01	570	1	570	1	93	1	565.83	-384.82	181.01
TCGA-C5-A1BI-01	1112	0	1112	0	1112	0	1119.3	-420.04	699.26
TCGA-C5-A1BJ-01	4385	0	4385	0	4385	0	654.09	-632.88	21.21
TCGA-C5-A1BK-01	5385	0	5385	0	5385	0	1919.23	-753.51	1165.71
TCGA-C5-A1BL-01	5271	0	5271	0	5271	0	750.93	-1367.78	-616.86
TCGA-C5-A1BM-01	2520	1	2520	0	2520	0	1358.64	-560.06	798.59
TCGA-C5-A1BN-01	166	1	166	1	166	1	-650.81	-1829.1	-2479.91
TCGA-C5-A1BQ-01	604	1	604	1	604	0	947.53	-1078.13	-130.59
TCGA-C5-A1M5-01	2052	1	2052	1	693	1	740.91	-1068.87	-327.96
TCGA-C5-A1M6-01	955	1	955	1	316	1	-938.2	-1597.75	-2535.94
TCGA-C5-A1M7-01	1409	0	1409	0	1409	0	592.08	-1105.16	-513.08
TCGA-C5-A1M8-01	919	0	919	0	919	0	-462.22	-1427.61	-1889.83
TCGA-C5-A1M9-01	1065	1	1065	1	555	1	-258.3	-1609.94	-1868.24
TCGA-C5-A1ME-01	1756	0	1756	0	1756	0	398.24	-1363.33	-965.08
TCGA-C5-A1MF-01	1617	0	1617	0	1617	0	774.56	-873.39	-98.84
TCGA-C5-A1MH-01	1186	1	1186	1	178	1	1487.7	-188.34	1299.36
TCGA-C5-A1MI-01	1083	1	1083	1	805	1	67.2	-991.29	-924.08
TCGA-C5-A1MJ-01	14	1	14	0	14	0	-5.79	1.69	-4.1
TCGA-C5-A1MK-01	74	1	74	1	74	1	200.57	-117.99	82.58
TCGA-C5-A1ML-01	636	1	636	1	636	1	18.13	-1121.33	-1103.19
TCGA-C5-A1MN-01	1245	1	1245	0	1245	0	-116.79	-1543.52	-1660.31
TCGA-C5-A1MP-01	109	0	109	0	109	0	1463.49	209.82	1673.31
TCGA-C5-A1MQ-01	1031	0	1031	0	1031	0	1579.58	-141.6	1437.98
TCGA-C5-A2LS-01	1345	0	1345	0	1345	0	-711.81	-2297.84	-3009.65
TCGA-C5-A2LT-01	2226	0	2226	0	2226	0	639.91	-477.45	162.46
TCGA-C5-A2LV-01	2234	0	2234	0	2234	0	799.58	-826.7	-27.12
TCGA-C5-A2LX-01	2526	0	2526	0	2526	0	1823.56	-513.88	1309.68
TCGA-C5-A2LY-01	2383	0	2383	0	2383	0	1811.68	-858.07	953.61
TCGA-C5-A2LZ-01	3046	1	3046	1	2893	1	464.77	-1616.62	-1151.85
TCGA-C5-A2M1-01	1169	0	1169	0	1169	0	786.33	-1478.94	-692.61
TCGA-C5-A2M2-01	1011	1	1011	1	1011	0	-523.07	-2048.53	-2571.6
TCGA-C5-A3HD-01	1582	0	1582	0	1582	0	-603.84	-2173.99	-2777.83
TCGA-C5-A3HE-01	548	0	548	0	252	1	1102.02	-961.49	140.53
TCGA-C5-A3HF-01	543	1	543	1	450	1	223.87	-917.71	-693.84
TCGA-C5-A3HL-01	621	0	621	0	621	0	-154.48	-1473.26	-1627.74

TCGA-C5-A7CG-01	6408	0	6408	0	6408	0	2234.92	-602.49	1632.43
TCGA-C5-A7CH-01	4694	0	4694	0	4694	0	-203.37	-1137.44	-1340.81
TCGA-C5-A7CJ-01	3097	1	3097	0	3097	0	982.59	0.05	982.64
TCGA-C5-A7CK-01	4086	1	4086	0	4086	0	-1009.79	-639.08	-1648.87
TCGA-C5-A7CL-01	471	1	471	1	471	1	36.07	-868.57	-832.5
TCGA-C5-A7CM-01	619	0	619	0	619	0	-670.58	-1038.37	-1708.95
TCGA-C5-A7CO-01	4482	0	4482	0	4482	0	70.1	-1684.63	-1614.53
TCGA-C5-A7UC-01	523	1	523	0	523	0	-602.9	-1590.67	-2193.57
TCGA-C5-A7UE-01	4738	0	4738	0	4738	0	-76.15	-1770.76	-1846.92
TCGA-C5-A7UH-01	3988	0	3988	0	3988	0	110.37	-967.77	-857.4
TCGA-C5-A7UI-01	2888	1	2888	0	2888	0	1220.77	-863.15	357.62
TCGA-C5-A7X3-01	284	1	284	0	284	0	709.79	-475.57	234.23
TCGA-C5-A7X5-01	414	1	414	1	414	1	-760.59	-1448.9	-2209.5
TCGA-C5-A7X8-01	83	0	83	0	83	0	188.49	60.89	249.38
TCGA-C5-A7XC-01	1551	0	1551	0	1551	0	761.17	-628.62	132.55
TCGA-C5-A8XH-01	1394	1	1394	0	1394	0	644.11	-1111.75	-467.64
TCGA-C5-A8XI-01	254	0	254	0	254	0	420.01	-1210.75	-790.74
TCGA-C5-A8XJ-01	4467	0	4467	0	4467	0	-146.47	-379.08	-525.55
TCGA-C5-A8XK-01	3039	0	3039	0	3039	0	29.51	-1435.66	-1406.15
TCGA-C5-A8YQ-01	715	1	715	1	715	1	4.29	-1328.65	-1324.37
TCGA-C5-A8YR-01	837	1	837	1	272	1	1051.58	-848.62	202.96
TCGA-C5-A8YT-01	633	1	633	1	633	1	-317.42	44.25	-273.17
TCGA-C5-A8ZZ-01	636	0	636	0	636	0	-672.83	167.85	-504.98
TCGA-C5-A901-01	518	0	518	0	518	0	148.26	-882.49	-734.23
TCGA-C5-A902-01	149	0	149	0	149	0	1054.41	-1642.05	-587.64
TCGA-C5-A905-01	4879	0	4879	0	4879	0	-313.27	-1951.57	-2264.83
TCGA-C5-A907-01	448	0	448	0	448	0	472	-1246.39	-774.39
TCGA-DG-A2KH-01	34	0	34	0	34	0	-712.91	-2586.99	-3299.9
TCGA-DG-A2KJ-01	2893	0	2893	0	2893	0	1431.35	-891.75	539.6
TCGA-DG-A2KK-01	2496	0	2496	0	2496	0	136.77	-1437.54	-1300.77
TCGA-DG-A2KL-01	1367	0	1367	0	1367	0	533.05	289.86	822.91
TCGA-DG-A2KM-01	1946	0	1946	0	1946	0	3295.3	448.78	3744.09
TCGA-DR-A0ZL-01	2669	0	2669	0	2669	0	-369.81	-1913.88	-2283.69
TCGA-DR-A0ZM-01	1791	0	1791	0	1791	0	1474.12	-576.81	897.31
TCGA-DS-A0VK-01	1118	1	1118	1	209	1	-467.26	-977.5	-1444.75
TCGA-DS-A0VL-01	81	0	81	0	81	0	421.27	-1294.91	-873.64
TCGA-DS-A0VM-01	3589	0	3589	0	868	1	275.77	-724.93	-449.16
TCGA-DS-A0VN-01	3609	0	3609	0	3609	0	1738.23	-258.07	1480.16
TCGA-DS-A1O9-01	266	1	266	1	266	1	1235.46	-155.63	1079.83
TCGA-DS-A1OA-01	879	1	879	1	379	1	893.89	-585.02	308.88
TCGA-DS-A1OB-01	861	1	861	1	652	1	240.77	-998.68	-757.92
TCGA-DS-A1OC-01	376	0	376	0	376	0	567.91	-1267.71	-699.8
TCGA-DS-A1OD-01	3874	0	3874	0	3874	0	1959.29	-406.09	1553.2
TCGA-DS-A3LQ-01	699	0	699	0	699	0	52.97	-260.26	-207.29
TCGA-DS-A5RQ-01	512	0	512	0	512	0	619.35	-1147.8	-528.45
TCGA-DS-A7WF-01	492	1	492	1	321	1	-415.17	-967.77	-1382.94

TCGA-DS-A7WH-01	533	0	533	0	533	0	-354.94	-93.15	-448.08
TCGA-DS-A7WI-01	252	1	252	1	206	1	374.32	-1640.55	-1266.23
TCGA-EA-A1QS-01	1203	0	1203	0	1203	0	141.82	778.01	919.83
TCGA-EA-A1QT-01	1243	0	1243	0	1243	0	-56.53	-1301.4	-1357.93
TCGA-EA-A3HQ-01	1136	0	1136	0	1136	0	906.86	-573.27	333.59
TCGA-EA-A3HR-01	940	0	940	0	940	0	-220.75	-1200.37	-1421.12
TCGA-EA-A3HS-01	959	0	959	0	959	0	227.86	-968.28	-740.42
TCGA-EA-A3HT-01	954	0	954	0	954	0	403.66	-43.86	359.8
TCGA-EA-A3HU-01	1013	0	1013	0	1013	0	1927.26	-717.38	1209.87
TCGA-EA-A3QD-01	397	0	397	0	397	0	2291.2	118.23	2409.43
TCGA-EA-A3QE-01	761	0	761	0	761	0	1681.67	-768.5	913.16
TCGA-EA-A3Y4-01	1122	0	1122	0	1122	0	1829.82	-759.72	1070.09
TCGA-EA-A410-01	803	0	803	0	803	0	-361.49	-403.06	-764.56
TCGA-EA-A411-01	747	0	747	0	747	0	108.83	38.96	147.79
TCGA-EA-A439-01	965	0	965	0	316	1	-388.75	-90.4	-479.14
TCGA-EA-A43B-01	791	0	791	0	791	0	997.44	-775.09	222.35
TCGA-EA-A44S-01	369	0	369	0	369	0	604.73	-938.41	-333.67
TCGA-EA-A4BA-01	755	0	755	0	755	0	-1336.18	-760.21	-2096.38
TCGA-EA-A50E-01	227	1	227	1	208	1	303.53	190.55	494.08
TCGA-EA-A556-01	453	0	453	0	453	0	-978.48	-261.55	-1240.03
TCGA-EA-A5FO-01	815	0	815	0	815	0	-833.21	-2400.89	-3234.1
TCGA-EA-A5O9-01	788	0	788	0	788	0	223.55	-1611.35	-1387.8
TCGA-EA-A5ZD-01	830	0	830	0	830	0	917.05	-615.87	301.18
TCGA-EA-A5ZE-01	829	0	829	0	425	1	1020.91	-178.95	841.96
TCGA-EA-A5ZF-01	828	0	828	0	440	1	-831.48	-555.64	-1387.12
TCGA-EA-A6QX-01	730	0	730	0	730	0	584.97	-1051.79	-466.82
TCGA-EA-A78R-01	410	0	410	0	410	0	535.4	-498.48	36.92
TCGA-EA-A97N-01	11	0	11	0	11	0	-199.19	-419.36	-618.55
TCGA-EK-A2GZ-01	383	0	383	0	383	0	-4.56	-1630.63	-1635.19
TCGA-EK-A2H0-01	1847	0	1847	0	1847	0	69.62	-1926.54	-1856.92
TCGA-EK-A2H1-01	799	0	799	0	799	0	990.9	-349.6	641.3
TCGA-EK-A2IP-01	202	0	202	0	202	0	-20.12	-1626.38	-1646.5
TCGA-EK-A2IR-01	3442	0	3442	0	3442	0	-1356.39	-1824.22	-3180.61
TCGA-EK-A2PG-01	46	0	46	0	46	0	-417.67	-2216.59	-2634.25
TCGA-EK-A2PI-01	586	0	586	0	586	0	-770.69	-943.04	-1713.73
TCGA-EK-A2PK-01	12	0	12	0	12	0	1554.9	-530.14	1024.75
TCGA-EK-A2PL-01	13	0	13	0	13	0	-68.25	-1722	-1790.25
TCGA-EK-A2PM-01	18	0	18	0	18	0	-1485.7	-1551.77	-3037.46
TCGA-EK-A2R7-01	27	0	27	0	27	0	355.32	-1088.56	-733.25
TCGA-EK-A2R8-01	44	0	44	0	44	0	-34.74	-1168.93	-1203.67
TCGA-EK-A2R9-01	4	0	4	0	4	0	783.87	-229.01	554.86
TCGA-EK-A2RA-01	1246	0	1246	0	1246	0	1124.97	-1605.84	-480.87
TCGA-EK-A2RB-01	9	0	9	0	9	0	51.18	-1634.24	-1583.06
TCGA-EK-A2RC-01	129	0	129	0	129	0	970.36	-547.74	422.61
TCGA-EK-A2RE-01	57	0	57	0	57	0	-662.56	-1428.04	-2090.6
TCGA-EK-A2RJ-01	53	0	53	0	53	0	1021.35	-904.95	116.4

TCGA-EK-A2RK-01	13	0	13	0	13	0	684.31	-1394.51	-710.2
TCGA-EK-A2RL-01	1453	1	1453	1	1143	1	-651.28	-1676	-2327.28
TCGA-EK-A2RM-01	50	0	50	0	50	0	-433.02	-513.48	-946.5
TCGA-EK-A2RN-01	71	0	71	0	71	0	1007.65	-1123.43	-115.78
TCGA-EK-A2RO-01	2	0	2	0	2	0	-167.77	-408.45	-576.22
TCGA-EK-A3GJ-01	3	0	3	0	3	0	1121.63	-1225.9	-104.27
TCGA-EK-A3GK-01	15	0	15	0	15	0	9.61	-2129.85	-2120.24
TCGA-EK-A3GN-01	27	0	27	0	27	0	-863.63	-2077.66	-2941.29
TCGA-EX-A1H5-01	619	0	619	0	619	0	-419.16	-1437.88	-1857.04
TCGA-EX-A1H6-01	241	0	241	0	241	0	-464.93	-1196.1	-1661.04
TCGA-EX-A3L1-01	463	0	463	0	463	0	593.01	-691.76	-98.75
TCGA-EX-A449-01	447	0	447	0	447	0	-583.35	-2035.14	-2618.49
TCGA-EX-A69L-01	602	0	602	0	602	0	1673.59	-531.43	1142.16
TCGA-EX-A69M-01	253	1	253	0	77	1	1155.32	-1293.85	-138.53
TCGA-EX-A8YF-01	473	0	473	0	396	1	908.4	-1170.83	-262.43
TCGA-FU-A23K-01	372	0	372	0	372	0	921.65	-602.15	319.5
TCGA-FU-A23L-01	725	0	725	0	725	0	995.75	-1146.42	-150.67
TCGA-FU-A2QG-01	579	0	579	0	579	0	-8.26	-1941.24	-1949.5
TCGA-FU-A3EO-01	490	0	490	0	490	0	305.91	-1094.7	-788.79
TCGA-FU-A3HY-01	954	0	954	0	954	0	-575.44	-1872.04	-2447.48
TCGA-FU-A3HZ-01	1103	0	1103	0	1103	0	-472.77	-1209.26	-1682.04
TCGA-FU-A3NI-01	638	1	638	0	638	0	79.64	-792.78	-713.14
TCGA-FU-A3TQ-01	795	0	795	0	795	0	390.51	-991.25	-600.74
TCGA-FU-A3TX-01	45	0	45	0	45	0	330.79	-1209.18	-878.39
TCGA-FU-A3WB-01	491	0	491	0	491	0	-587.04	-1392.12	-1979.16
TCGA-FU-A3YQ-01	861	0	861	0	861	0	1299.46	-131.85	1167.61
TCGA-FU-A40J-01	426	0	426	0	426	0	257.46	-1020.16	-762.7
TCGA-FU-A57G-01	1078	0	1078	0	1078	0	-573.87	307.4	-266.47
TCGA-FU-A5XV-01	321	0	321	0	321	0	-34.78	-1381.33	-1416.11
TCGA-FU-A770-01	34	0	34	0	34	0	-177.31	-1641.46	-1818.77
TCGA-GH-A9DA-01	540	0	540	0	540	0	504.42	-976.51	-472.1
TCGA-HG-A2PA-01	773	1	773	1	773	1	301.3	-767.43	-466.13
TCGA-HM-A3JJ-01	659	1	659	1	385	1	610.74	-147.22	463.52
TCGA-HM-A3JK-01	632	0	632	0	632	0	560.24	539.2	1099.44
TCGA-HM-A4S6-01	454	0	454	0	454	0	1119.15	-400.09	719.06
TCGA-HM-A6W2-01	287	0	287	0	287	0	-275.29	-528.24	-803.52
TCGA-HM-A6W2-06	287	0	287	0	287	0	-1675.65	-1212.71	-2888.36
TCGA-IR-A3L7-01	4483	0	4483	0	4483	0	-865.74	-1437.79	-2303.53
TCGA-IR-A3LA-01	4172	0	4172	0	4172	0	-247.07	-992.81	-1239.89
TCGA-IR-A3LB-01	2032	1	2032	1	317	1	-139.68	-95.4	-235.08
TCGA-IR-A3LC-01	3935	0	3935	0	3935	0	140.4	-1264.31	-1123.91
TCGA-IR-A3LF-01	2949	0	2949	0	2949	0	-0.07	-1092.11	-1092.18
TCGA-IR-A3LH-01	2394	0	2394	0	2394	0	2249.21	451.87	2701.08
TCGA-IR-A3LI-01	2493	0	2493	0	2493	0	-732.59	-2544.49	-3277.08
TCGA-IR-A3LK-01	908	1	908	1	574	1	506.07	-1565.59	-1059.51
TCGA-IR-A3LL-01	1106	0	1106	0	1106	0	1824.7	-790.52	1034.19

TCGA-JW-A5VG-01	834	0	834	0	834	0	642.09	-1403.27	-761.18
TCGA-JW-A5VH-01	100	1	100	1	94	1	-1434.35	-894.64	-2329
TCGA-JW-A5VI-01	747	0	747	0	558	1	448.26	-573.11	-124.84
TCGA-JW-A5VJ-01	652	0	652	0	267	1	-159.99	-1449.23	-1609.22
TCGA-JW-A5VK-01	623	0	623	0	623	0	-552.07	-1883.09	-2435.15
TCGA-JW-A5VL-01	474	0	474	0	474	0	1044.4	-1175.58	-131.18
TCGA-JW-A69B-01	863	0	863	0	863	0	246.57	-893.62	-647.05
TCGA-JW-A852-01	252	1	252	1	153	1	246.99	-1672.36	-1425.36
TCGA-JW-AAVH-01	552	0	552	0	552	0	-422.62	-1900.07	-2322.69
TCGA-JX-A3PZ-01	642	1	642	1	491	1	1000.74	-566.59	434.15
TCGA-JX-A3Q0-01	6375	0	6375	0	6375	0	918.68	-1201.57	-282.9
TCGA-JX-A3Q8-01	1357	0	1357	0	1357	0	402.29	-1466.49	-1064.21
TCGA-JX-A5QV-01	636	0	636	0	636	0	401.17	-1499.45	-1098.28
TCGA-LP-A4AU-01	343	0	343	0	343	0	267.33	-1067.32	-799.99
TCGA-LP-A4AW-01	27	0	27	0	27	0	-191.83	38.09	-153.74
TCGA-LP-A4AX-01	380	0	380	0	380	0	1471.42	-448.6	1022.82
TCGA-LP-A5U2-01	9	0	9	0	9	0	-228.17	-1568.41	-1796.57
TCGA-LP-A5U3-01	25	0	25	0	25	0	489.92	-1451.04	-961.12
TCGA-LP-A7HU-01	406	0	406	0	406	0	606.3	-840	-233.7
TCGA-MA-AA3W-01	685	0	685	0	685	0	1800.25	-1035.83	764.42
TCGA-MA-AA3X-01	617	0	617	0	617	0	900.24	-474.99	425.25
TCGA-MA-AA3Y-01	542	0	542	0	542	0	1274.81	-583.19	691.62
TCGA-MA-AA3Z-01	595	0	595	0	595	0	711.59	-1728.17	-1016.58
TCGA-MA-AA41-01	279	0	279	0	279	0	61.14	-1511.67	-1450.53
TCGA-MA-AA42-01	259	0	259	0	259	0	2651.87	-656.81	1995.06
TCGA-MA-AA43-01	346	0	346	0	346	0	-268.53	-461.87	-730.39
TCGA-MU-A51Y-01	854	0	854	0	854	0	1435.19	-313.82	1121.38
TCGA-MU-A5YI-01	1053	0	1053	0	630	1	410.04	200.51	610.54
TCGA-MU-A8JM-01	607	0	607	0	607	0	547.59	-1211.23	-663.64
TCGA-MY-A5BD-01	1667	0	1667	0	1667	0	-582.83	-1880.91	-2463.74
TCGA-MY-A5BE-01	1066	0	1066	0	1066	0	3002.07	-107.98	2894.09
TCGA-MY-A5BF-01	634	0	634	0	634	0	847.99	-1267.4	-419.41
TCGA-MY-A913-01	524	0	524	0	524	0	550.82	-1059.36	-508.54
TCGA-PN-A8MA-01	90	0	90	0	90	0	41.35	-734.6	-693.25
TCGA-Q1-A5R1-01	474	0	474	0	474	0	-197.06	-1681.01	-1878.07
TCGA-Q1-A5R2-01	499	0	499	0	499	0	999.15	-643.54	355.61
TCGA-Q1-A5R3-01	485	0	485	0	485	0	689.36	-1461.12	-771.76
TCGA-Q1-A6DT-01	275	1	275	1	275	1	-375.58	-1370.25	-1745.83
TCGA-Q1-A6DV-01	491	0	491	0	491	0	-435.35	-1903.08	-2338.43
TCGA-Q1-A6DW-01	534	0	534	0	534	0	23.55	-1155.96	-1132.41
TCGA-Q1-A73O-01	428	0	428	0	428	0	495.99	-1019.3	-523.31
TCGA-Q1-A73P-01	483	0	483	0	483	0	231.42	-1273.21	-1041.79
TCGA-Q1-A73Q-01	284	0	284	0	284	0	-527.03	-1501.81	-2028.83
TCGA-Q1-A73R-01	567	0	567	0	567	0	-732.66	-1213.9	-1946.55
TCGA-Q1-A73S-01	688	0	688	0	688	0	-1038.39	-1569.61	-2608
TCGA-R2-A69V-01	596	0	596	0	596	0	2825.15	66.92	2892.07

TCGA-RA-A741-01	444	0	444	0	444	0	1027.72	-853.84	173.88
TCGA-UC-A7PD-01	355	1	355	1	202	1	-99.43	-1425.5	-1524.93
TCGA-UC-A7PF-01	2859	1	2859	1	2859	0	1420.43	-449.24	971.19
TCGA-UC-A7PG-01	370	1	370	1	370	1	-244.68	-916.06	-1160.74
TCGA-UC-A7PG-06	370	1	370	1	370	1	-949.19	1003.99	54.8
TCGA-UC-A7PI-01	2114	0	2114	0	778	1	-514.89	-1853.9	-2368.78
TCGA-VS-A8EB-01	305	1	305	1	305	1	372.05	-1202.96	-830.91
TCGA-VS-A8EC-01	1415	0	1415	0	1415	0	-1529.91	-2188	-3717.91
TCGA-VS-A8EG-01	1386	0	1386	0	1386	0	-1916.32	-221.07	-2137.39
TCGA-VS-A8EH-01	986	0	986	0	986	0	-405.97	-1861.15	-2267.12
TCGA-VS-A8EI-01	729	0	729	0	729	0	663.64	-877.92	-214.28
TCGA-VS-A8EJ-01	607	1	607	1	607	1	-241.6	-1464.79	-1706.39
TCGA-VS-A8EK-01	829	1	829	1	829	1	-54.59	-1764.26	-1818.85
TCGA-VS-A8EL-01	1992	0	1992	0	1992	0	1610.41	-539.58	1070.84
TCGA-VS-A8Q8-01	978	1	978	1	488	1	991.86	-1041.04	-49.18
TCGA-VS-A8Q9-01	1630	0	1630	0	1630	0	-148.44	-1803.68	-1952.12
TCGA-VS-A8QA-01	1099	0	1099	0	1099	0	-91.03	-2262.94	-2353.97
TCGA-VS-A8QC-01	350	1	350	1	263	1	-92.79	-677.22	-770.01
TCGA-VS-A8QF-01	1800	0	1800	0	1800	0	581.76	-913.09	-331.34
TCGA-VS-A8QH-01	1210	1	1210	1	1210	1	-521.78	-1643.91	-2165.69
TCGA-VS-A8QM-01	951	1	951	1	951	1	1685.8	-92.6	1593.2
TCGA-VS-A94W-01	1243	0	1243	0	1243	0	1356.47	-811.52	544.94
TCGA-VS-A94X-01	506	1	506	1	506	1	-26.12	-1715.48	-1741.6
TCGA-VS-A94Y-01	144	1	144	1	144	1	188.05	-826.27	-638.22
TCGA-VS-A94Z-01	1015	0	1015	0	1015	0	1900.48	-563.79	1336.69
TCGA-VS-A950-01	1221	0	1221	0	1221	0	-135.41	-1093.35	-1228.75
TCGA-VS-A952-01	1778	0	1778	0	1778	0	-487.8	-1896.65	-2384.44
TCGA-VS-A953-01	477	1	477	1	477	1	30.24	-1807.81	-1777.57
TCGA-VS-A954-01	1714	0	1714	0	1714	0	-86.51	-962.79	-1049.3
TCGA-VS-A957-01	1688	0	1688	0	1688	0	-483.76	-2241.63	-2725.39
TCGA-VS-A958-01	1525	0	1525	0	1525	0	1872.28	-971.71	900.57
TCGA-VS-A959-01	1561	0	1561	0	1561	0	572.52	-1022.6	-450.08
TCGA-VS-A9U5-01	1535	0	1535	0	1535	0	555.39	-1459.98	-904.59
TCGA-VS-A9U6-01	1320	0	1320	0	711	1	459.65	-924.95	-465.3
TCGA-VS-A9U7-01	1472	0	1472	0	1472	0	992.56	-1087.85	-95.29
TCGA-VS-A9UB-01	911	0	911	0	911	0	1772.84	-1043.92	728.92
TCGA-VS-A9UC-01	825	0	825	0	825	0	608.45	-645.13	-36.68
TCGA-VS-A9UD-01	739	0	739	0	739	0	1998.04	-1246.25	751.8
TCGA-VS-A9UH-01	1427	0	1427	0	1427	0	1967.06	195.31	2162.37
TCGA-VS-A9UI-01	1372	1	1372	0	1372	0	688.03	-1510.64	-822.6
TCGA-VS-A9UJ-01	52	1	52	1	52	1	-1362.79	-1608.69	-2971.48
TCGA-VS-A9UL-01	442	1	442	1	336	1	-1645.63	-1997.61	-3643.23
TCGA-VS-A9UM-01	829	1	829	0	829	0	821.17	-313.69	507.48
TCGA-VS-A9UO-01	1456	0	1456	0	1456	0	-366.6	-2568.14	-2934.73
TCGA-VS-A9UP-01	1444	0	1444	0	352	1	-1556.77	-1406.97	-2963.73
TCGA-VS-A9UQ-01	1263	0	1263	0	561	1	-484.1	-906.09	-1390.2

TCGA-VS-A9UR-01	793	0	793	0	675	1	-567.35	-1724.64	-2291.99
TCGA-VS-A9UT-01	482	0	482	0	482	0	2086.32	91.42	2177.73
TCGA-VS-A9UU-01	442	0	442	0	442	0	-340.02	-1710.18	-2050.19
TCGA-VS-A9UV-01	104	1	104	1	104	1	-259.79	-1725.07	-1984.87
TCGA-VS-A9UY-01	555	1	555	1	412	1	411.84	-1157.41	-745.57
TCGA-VS-A9UZ-01	2044	0	2044	0	2044	0	-30.82	-1425.62	-1456.43
TCGA-VS-A9V0-01	573	0	573	0	475	1	-1007.2	-1698.04	-2705.24
TCGA-VS-A9V1-01	157	1	157	1	58	1	-109.84	-1848.97	-1958.81
TCGA-VS-A9V2-01	555	0	555	0	555	0	916.97	-978.46	-61.49
TCGA-VS-A9V3-01	540	0	540	0	456	1	655.03	-1145.48	-490.45
TCGA-VS-A9V4-01	132	1	132	1	100	1	-641.97	-1860.3	-2502.27
TCGA-VS-A9V5-01	494	1	494	1	494	1	-623.29	-1440.48	-2063.77
TCGA-VS-AA62-01	469	1	469	1	469	1	1553.28	-635.91	917.36
TCGA-WL-A834-01	791	0	791	0	791	0	-546.46	-1208.95	-1755.41
TCGA-XS-A8TJ-01	890	0	890	0	890	0	367.43	-784.4	-416.97
TCGA-ZJ-A8QQ-01	2056	0	2056	0	2056	0	26.65	-1687.59	-1660.94
TCGA-ZJ-A8QR-01	582	1	582	1	582	0	76.9	-1631.87	-1554.96
TCGA-ZJ-AAX4-01	21	0	21	0	21	0	1194.43	-576.56	617.86
TCGA-ZJ-AAXA-01	43	0	43	0	43	0	-450.39	-1109.28	-1559.67
TCGA-ZJ-AAXU-01	5	0	5	0	5	0	972.84	-1206.4	-233.56
TCGA-ZX-AA5X-01	119	0	119	0	119	0	960.31	524.51	1484.82
